# Natural diversity in phenolic components and antioxidant properties of oregano (*Origanum vulgare* L.) accessions, grown under the same conditions

**DOI:** 10.1038/s41598-022-09742-4

**Published:** 2022-04-06

**Authors:** Ghazaleh Jafari Khorsand, Mohammad Reza Morshedloo, Hasan Mumivand, Zohreh Emami Bistgani, Filippo Maggi, Abdolvahab Khademi

**Affiliations:** 1 Department of Horticultural Sciences, Faculty of Agriculture, University of Maragheh, Maragheh, Iran; 2 Department of Horticultural Sciences, Faculty of Agriculture, Lorestan University, Khorramabad, Iran; 3 Agricultural Research Education and Extension Organization (AREEO), Isfahan Agricultural and Natural Resources Research and Education Center, Isfahan, Iran; 4 School of Pharmacy, Chemistry Interdisciplinary Project (ChIP), University of Camerino, Camerino, Italy; 5 Department of Mathematics and Statistics, University of Massachusetts Amherst, Amherst, USA

**Keywords:** Biochemistry, Plant sciences

## Abstract

Oregano (*Origanum vulgare* L.) is a rich source of biologically active components such as phenolic compounds. Here, seven pot grown *O. vulgare* accessions belonging to three subspecies (subsp. *virens*, subsp. *vulgare* and subsp. *gracile*) were investigated for their content in sixteen bioactive phenolic compounds as well as their antioxidant capacities (DPPH^•^ and FRAP tests), total phenolic content (TPC) and total flavonoid content (TFC) in order to identify the most suitable ones on an industrial level. HPLC analyses showed that rosmarinic acid (659.6–1646.9 mg/100 g DW) was by far the most abundant constituent, followed by luteolin (46.5–345.4 mg/100 g DW), chicoric acid (36.3–212.5 mg/100 g DW), coumarin (65.7–193.9 mg/100 g DW) and quercetin (10.6–106.1 mg/100 g DW), with variability in concentration depending on the accession and subspecies. The highest level of rosmarinic acid and TPC was obtained from Ardabil accession (subsp. *virens*). There was a significant and positive correlation between rosmarinic acid and antioxidant activity (r = 0.46). TFC significantly correlated to TPC (r = 0.57) as well as to chicoric acid (r = 0.73). Cluster (CA) and principal component (PCA) analyses classified the investigated accessions in three different groups. Such natural variabilities in phenolics provide the possibility of using elite plants for nutraceutical and pharmaceutical industries and domestication of highly antioxidative accessions of oregano.

## Introduction

Herbs represent an extensive variety of aromatic plants utilized for food flavoring and therapeutic goals^1^. The fragrances, flavors, and therapeutic characteristics of herbs are related to their secondary metabolites^2^. These chemicals, mostly within the essential oil or extract, are synthesized in the plant during the course of metabolism^3^. The genus *Origanum* (Lamiaceae) includes some well-known annual, perennial, and shrubby herbs with wide morphological and chemotype diversity throughout the world^4,5^. Most types of the genus are distributed in the Mediterranean, Euro-Siberian, and Iran-Siberian regions^1^. *Origanum vulgare* L., commonly known as oregano, is generally recognized as a renowned condiment and culinary herb in the world^1^. Based on the recent classification of the World Flora Online (WFO; www.worldfloraonline.org), five different subspecies have been reported for *O. vulgare* (i.e. subsp. *glandulosum*, subsp. *gracile*, subsp. *hirtum*, subsp. *virens* and subsp. *vulgare*). Of these, the subsp. *vulgare*, *gracile* and *virens* are wildly distributed in Iran^6^. Among the mentioned Iranian subspecies, *O. vulgare* subsp. *gracile* is a rich source of phenolic monoterpenes such as carvacrol^7^. The aerial parts of oregano contain a wide array of chemical constituents, including phenolics, terpenes, flavonoids, glucosides, sterols, tannins, resins and mucilages^1,8,9^. The plant is commonly used as a flavoring herb in food production and industry and to flavor salads, soups, fish, pizza, processed meats, and other eatables^9^. In addition, oregano, as a medicinal plant, has been traditionally used as an expectorant, anti-flatulence, appetizer, diuretic and sedative agent^2^. The plant extract has strong antibacterial and antifungal properties due to its richness in phenolic compounds^6^. Rosmarinic acid, luteolin, quercetin, apigenin, scutellarein and their derivatives are the major phenolic acids and flavonoids that have been detected in oregano species^10^.

Through inhibiting the initiation of oxidizing chain reaction, antioxidants inhibit or delay the oxidation of molecules. Because of its high phenolic content, oregano is considered as one of the most favorite natural antioxidants^11^. The antioxidant capacity of *O*. *vulgare* extract is mainly related to its phenolic constituents^8,12^. The phenolic antioxidants take over various pharmacological properties, such as antidiabetic, antiulcer, antiviral, cytotoxic, antitumor and anti-inflammatory activities^13^, and are mainly responsible for the health effects of *O. vulgare*. Potential anticancer characteristics of phenolic acids and flavonoids have been previously reported by Shukla and Gupta (2010)^14^.

Our previous investigations showed that there is a great variability in terms of essential oil compounds among the different subspecies and/or accessions of oregano herb^5,15^. However, there is also a big challenge as to whether there is variation in phenolics and antioxidant capacity among the accessions and subspecies of oregano. A previous study has shown a high variability in rosmarinic acid content and oxygen radical absorbance capacity (ORAC) and total phenolic content (TPC) of European oregano accessions^11^. As a result, it can be presumed that such variability in rosmarinic acid and other phenolic compounds can also be seen in Iranian oregano accessions. Considering the great importance of phenolic compounds in human nutrition and their health effects, the current study aimed to investigate the variability in main phenolic compounds (including rosmarinic acid, chicoric acid, apigenin, luteolin and others), total phenolic content (TPC), total flavonoids contents (TFC) and antioxidant capacity among different accessions of Iranian oregano belonging to three subspecies (subsp. *virens*, subsp. *vulgare* and subsp. *gracile*). This research is a continuation of an ongoing oregano breeding program, pointing out the elite accessions of oregano in terms of phenolic components for domestication. In the meantime, the results generated from the current study will be useful to introduce the elite accessions with high antioxidant capacity into food and pharmaceutical industries.

## Materials and methods

### Reagents and standards

HPLC grade rosmarinic acid, chlorogenic acid, cinnamic acid, quercetin, caffeic acid, syringic acid, benzoic acid, vanillic acid, gallic acid, apigenin, chicoric acid, luteolin, kaempferol, 2,4-dihydroxybenzoic acid, naringenin and coumarin standards were purchased from Sigma-Aldrich (MO, USA). Other chemicals and solvents were analytical grade and were purchased from Merck (Darmstadt, Germany).

### Plant and soil materials

Seeds of the seven accessions of *O. vulgare* belonging to three subspecies (subsp. *virens*, subsp. *vulgare* and subsp. *gracile*) were obtained from the seed gene bank of the Research Institute of Forest and Rangeland in Tehran, Iran. The seed gene bank declared that seeds of all accessions were obtained under national and international guidelines and the seed were prepared under the supervision and permission of Maragheh University and all authors comply with all the local and national guidelines. The voucher specimens of the plants were deposited at the herbarium of Department of Horticultural Sciences, University of Tehran, Karaj, Iran. Geological characteristics of the seed, collection sites, subspecies and their voucher numbers are presented in Table 1.

**Table 1:** Origin, subspecies and geographical characteristics of oregano accessions.

Populations	Area of sampling collection	Subspecies	Longitude (E)	Latitude (N)	Altitude (m)	Voucher number
Baneh	Kordistan province—Baneh	*gracile*	45°59′	36°06	1750	6434
Mazandaran	Mazandaran province—Chalus	*virens*	51°22′	36°39′	20	6440
Gilan	Gilan province—Roudbar	*virens*	49°29′	36°51′	177	6439
Ardabil	Ardebil province—Ardabil	*virens*	48°15′	38°20′	1320	6436
Namin	Ardebil province—Aliabad	*virens*	48°30′	38°24′	1370	6435
Kaleybar	East Azerbaijan province—Kaleybar	*vulgare*	47°08′	38°54′	1360	6438
Arasbaran	East Azerbaijan province—Arasbaran	*vulgare*	46°53′	38°57′	950	6437

Oregano seeds were sown in a plastic germination tray filled with coco peat:perlite mixture (70:30, w:w) and kept in a glass greenhouse at the University of Maragheh, Maragheh, Iran. On 12 April 2020, the seedlings with about 10 cm height were transferred into 7 L pots. The growth medium was composed of combined proportions of field soil, silt, leaf mold and perlite (45:25:20:10, v:v). The soil contained 0.09% N, 21.8 mg kg^−1^ available P, and 487 mg kg^−1^ available K; the medium pH was 7.2 and EC was adjusted to be 1.2 dS m^−1^. During the growth seasons, the plants were irrigated regularly. The pots were placed in the greenhouse under natural daylight with a maximum and minimum day temperature of 33.5 and 17.5 °C, respectively. To warrant the well growth of the plants, the pots were fed with half Hoagland-based solution six times during the growth period (Jons, 2014). The harvest was performed at full flowering stage on 15 August 2020. The plants were cut from 5 cm above the soil and dried in oven at 40 °C, after which their dry drug weight (flowering aerial part) was measured (g/pot). For each accession, 12 individual plants were grown and each of the four harvested oregano plants was bulked together to obtain three replications (n = 3) for the extraction purpose.

### Plant extraction procedure

For extraction, 200 mg of leaf and inflorescence powder from each dried sample was dissolved in 20 mL of 80% methanol (methanol–water mixture in 80:20 proportion) and shaken for 72 h at room temperature (25 °C). Then, the supernatant was filtered using a Whatman filter paper (No. 4) and the residue was re-extracted with the same method^16^.

### Determination of total phenolic compounds

In order to determine the total phenolic content of oregano accessions, the Folin-Ciocalteu method was used (Spanos and Wrolstad 1990). For this purpose, 10 μL of plant extract were added to 500 μL of 10% Folin-Ciocalteu's reagent. Next, 500 μL of 1% saturated sodium carbonate solution were added to the mixture and incubated for 2 h at 25 °C. After incubation, the solution absorbance was read at 765 nm using a microplate reader (Spectromax-M5-USA). Gallic acid with different concentrations was used to make the calibration curve. The total phenolics content was reported as mg gallic acid equivalent (GAE) g^−1^ dry weight^17^.

### Determination of total flavonoids

According to the outline described by Quettier-Deleu et al. (2000)^18^, total flavonoids content of oregano extracts was measured using AlCl_3_ reagent. Briefly, 2 mL of 2% methanolic aluminum chloride solution was added to 2 mL of the extract and then stirred slightly. The procedure was continued by adding 6 mL of 5% potassium acetate solution. The mixture was incubated for 40 min at 25 °C and after that the absorbance of samples was read at 415 nm using a microplate reader (Spectromax-M5-USA). For quantification of absorbance, quercetin was used as a standard. The amount of flavonoids in each extract was expressed in terms of mg quercetin equivalent (QE) g^−1^ dry weight.

### RP-HPLC analysis of phenolic compounds

To identify the phenolic components, oregano extracts were analyzed using high-performance liquid chromatography (HPLC, Shimadzu, Japan) coupled with a photodiode array detector (SPD-M20A, Shimadzu, Japan). Phenolics were separated using a phenomenex column (Gemini-5 µm NX-C18 110 Å, LC Column 250 × 4.6 mm). The mobile phase was composed of solvent B (20 mM phosphoric acid) and solvent D (methanol). The program began with isocratic elution with 10% B (1.5 min), then a linear gradient was used until 40 min, increasing B to 95%, 5 min in 95% B, decreased to 10% in 30 s, 6 min in 10%. The flow rate was 1 mL min^−1^. Ten μL from each sample were injected into instrument. Scanning was performed from wavelength range of 200 to 400 nm. The identification of phenolic components was performed by comparing their pure standard retention time and the UV spectra with those of standards. For quantification of the components, different concentrations of each standard (10–80 μg mL^−1^) were injected under the same operative conditions of extracts and the calibration curve of each compound was plotted. The results were exposed as mg of each compound per 100 g of dry weight (mg/100 g DW).

### DPPH-radical scavenging activity

The method described by Chou et al., (2011)^8^ was used to capture DPPH (2,2-diphenyl-1-picrylhyrazyl) free radical scavenging activity of oregano extracts. Toward that end, different concentrations of oregano extract (10 μL) were added to 990 μL of 0.1 mM methanolic solution of DPPH. After 30 min of incubation under darkness at room temperature, the absorbance was recorded at 517 nm using a microplate reader (Spectromax-M5-USA). Ascorbic acid was used as a positive control. Ethanolic solution of DPPH was used as control. DPPH radical scavenging activity (%) = [(A _control_ – A _final DPPH_) / A _control_] × 100.

Linear regression analysis was used to determine the EC_50_ (half maximal effective concentration) value of the extracts.

### Ferric-reducing antioxidant power (FRAP) assay

Ferric-reducing antioxidant power (FRAP) of the extracts was determined using the method explained by Benzie and Strain (1996)^19^ with minor modifications. Following the procedure, the FRAP reagent was prepared by mixing 100 mL of acetate buffer (300 mM; pH 3.6), 10 mL of TPTZ (10 mM in HCl), and 10 mL of FeCl_3_.6H_2_O (20 mM in water). The fresh mixture was incubated at 37 °C until use. Acetate buffer was prepared by mixing 0.31 g of sodium acetate trihydrate with 1.6 mL of glacial acetic acid and reaching the volume of 100 mL using doubly distilled water. The reaction was done by mixing 380 μL of FRAP reagent and 20 μL of plant extracts in the wells and incubating at 37 °C for 10 min. The absorbance of samples was read at 593 nm using Spectromax-M5 microplate reader (USA). Quantification was done when the calibration curve of Fe_2_SO_4_.7H_2_O (25–1000 µM) was plotted. The FRAP antioxidant activity was expressed in terms of μM Fe (II).

### Statistical analysis

Analysis of variance (ANOVA) and least significant difference (LSD) test at a 5% probability level was performed using SAS statistical software (version 9.1, SAS Inst., USA). Pearson correlation coefficients were computed using IBM SPSS (SPSS, version 22, USA). Cluster analysis and principal component analysis (PCA) were performed using Xlstat software, 2018.

## Results

### Drug yield

**Figure 1:**
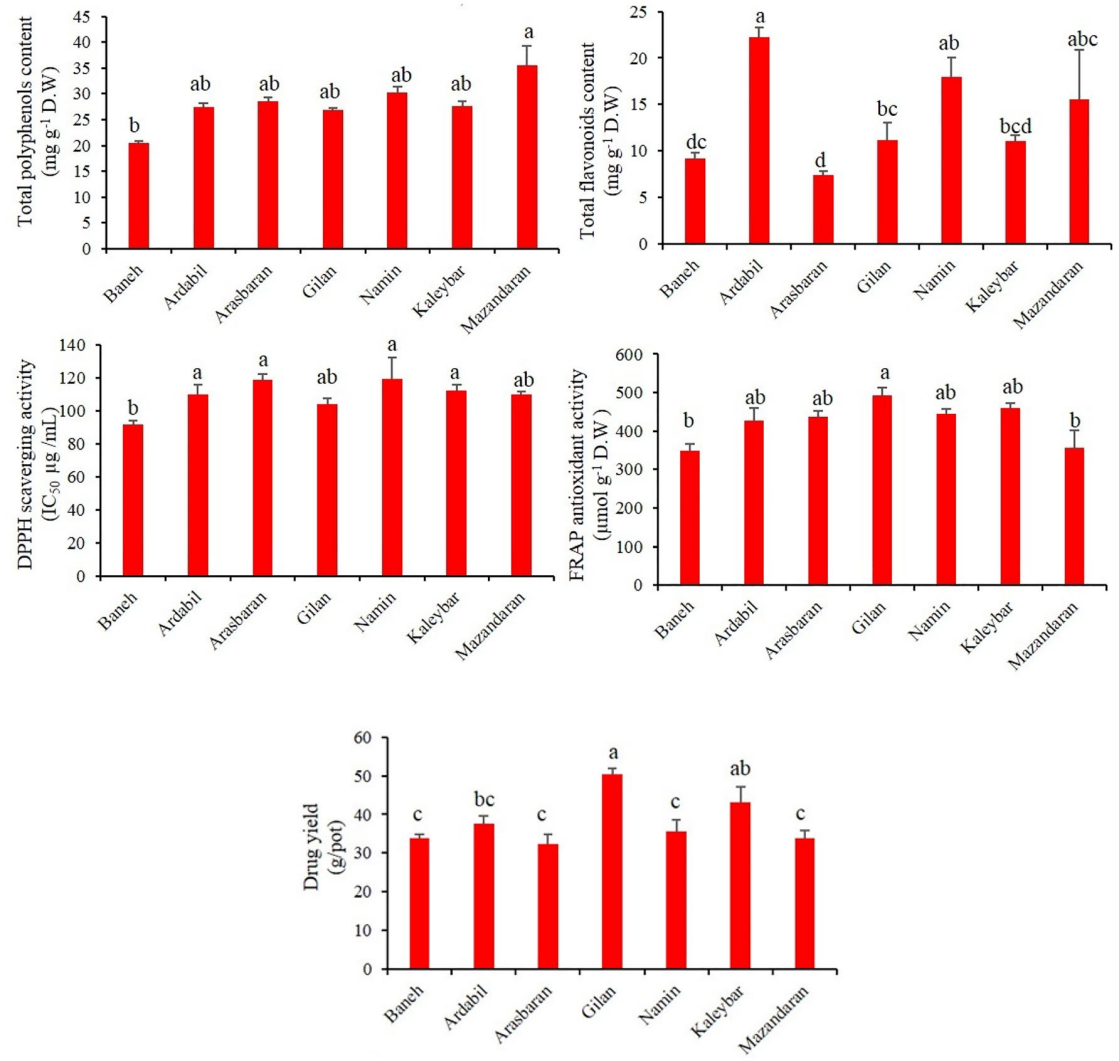
Mean values (n = 3) of yield and antioxidant biochemicals in seven oregano accessions. The error bars represent standard errors of the means. Different letters indicate significant differences (*p* < 0.05) among the treatments within each genotype (LSD test at 5% level).

The results from ANOVA showed that there were significant differences (p < 0.01) among oregano accessions in terms of drug yield (flowering aerial parts). As depicted in Fig. 1, a high variability was observed among the oregano accessions in terms of drug yield, ranging from 32.3 to 50.5 g/pot. According to mean compression values, the highest drug yield (50.5 g/pot) was obtained for Gilan accession (subsp. *virens*), followed by Kaleybar (43.2 g/pot; subsp. *vulgare*) and Ardabil (37.5 g/pot; subsp. *virens*) accessions. However, Arasbaran (32.3 g/pot; subsp. *vulgare*), Baneh (33.7 g/pot; subsp. *gracile*) and Mazandaran (33.8 g/pot; subsp. *virens*) accessions yielded the lowest drug weights in comparison with others.

### Total polyphenol and flavonoid contents

As depicted in Fig. 1, based on the mean compression of TPC in oregano accessions, a high variability was observed. According to the results, total polyphenols contents varied from 20.3 to 35.5 mg GAE g^−1^ DW. The highest and lowest TPC were observed in Mazandaran (subsp. *virens*) and Baneh (subsp. *gracile*) accessions, respectively. This creates a variance of roughly 44% between the lowest and highest concentration levels of TPC. However, there was no significant difference among the Ardabil, Arasbaran, Gilan, Namin and Kaleybar accessions in terms of TPC (Fig. 1). According to the ANOVA results, TFC were largely variable among the investigated accessions (*p* < 0.01). Overall, Ardabil and Arasbaran accessions had the highest and lowest TFC (22.2 and 7.7 mg QE g^−1^ DW, respectively) (Fig. 1). This creates about a three-fold variation among the mentioned accessions.

### Phenolic constituents

To identify the phenolic components in different oregano accessions, RP-HPLC analysis was performed. In total, sixteen major phenolic metabolites, including ten phenolic acids (rosmarinic, chlorogenic, cinnamic, caffeic, syringic, benzoic, vanillic, gallic, chicoric, and 2,4-dihydroxybenzoic acids), five flavonoids (quercetin, apigenin, luteolin, naringenin, kaempferol) and coumarin were identified. Table 2 illustrates the phenolic components of the oregano plants. Among the investigated phenolics, rosmarinic acid was identified as the major component in all extracts. According to the ANOVA results, significant differences (*p* < 0.01) were found among different oregano accessions and subspecies in terms of rosmarinic acid content. The maximum amount of rosmarinic acid (1646.9 ± 29.3 mg/100 g DW) was obtained from Ardebil accession (*O. vulgare* subsp. *virence*), while the minimum content (659.6 ± 66.6 mg/100 g DW) was found in Baneh population (*O. vulgare* subsp. *gracile*). Namin (1606.7 ± 120.0 mg/100 g DW), Arasbaran, (1484.2 ± 200.1 mg/100 g DW) and Kaleybar (1164.8 ± 28.6 mg/100 g DW) accessions also had a high amount of rosmarinic acid. Luteolin was the second major phenolic compound of oregano. The highest (345.4 ± 56.1 mg/100 g DW) and lowest (77 ± 42.1 mg/100 g DW) amount of luteolin were observed in Namin and Mazandaran accessions, respectively. Chicoric acid (ranging from 36 ± 3 to 212 ± 1.53 mg/100 g DW), coumarin (ranging from 65 ± 3 to 193.9 ± 8.9 mg/100 g DW), quercetin (ranging from 10 ± 3.9 to 106 ± 15.1 mg/100 g DW) and vanillic acid (ranging from 21.6 ± 8.4 to 89.7 ± 3.1 mg/100 g DW) were the other dominant phenolics detected in all accessions. The other phenolic components were recorded in lower amounts in all oregano plants.

**Table 2:** Phenolic components in extract of seven oregano accession ± standard deviation (SD).

Phenolic components (mg/100 g dry weight)	Oregano populations							
	Baneh	Ardabil	Arasbaran	Gilan	Namin	Kaleybar	Mazandaran	LSD^a^
Rosmarinic acid	659.6 ± 66.6	1646.9 ± 29.3	1484.2 ± 200.1	816.4 ± 104.1	1606.7 ± 120.0	1164.8 ± 28.6	967.7 ± 64.1	315.01
Chlorogenic acid	0.9 ± 0.4	0.8 ± 0.4	0.2 ± 0.1	1.1 ± 0.9	0.6 ± 0.3	0.9 ± 0.03	2.7 ± 0.1	1.61
Cinnamic acid	2.7 ± 1.9	1.8 ± 0.3	5.6 ± 1.8	7.1 ± 0.4	3.0 ± 1.0	10.8 ± 2.8	4.6 ± 0.4	4.41
Quercetin	106.0 ± 15.2	59.0 ± 3.7	10.6 ± 4.0	32.1 ± 16.5	22.9 ± 1.8	15.2 ± 1.3	40.6 ± 2.9	26.75
Caffeic acid	2.2 ± 0.3	1.2 ± 0.3	1.4 ± 0.2	0.4 ± 0.01	0.5 ± 0.1	2.2 ± 0.3	28.8 ± 2.7	3.92
Syringic acid	6.2 ± 1.0	8.0 ± 0.8	7.4 ± 0.7	6.8 ± 0.5	5.1 ± 2.1	7.4 ± 0.8	27.2 ± 0.6	7.3
Benzoic acid	10.9 ± 3.3	6.6 ± 0.7	11.6 ± 4.2	6.2 ± 0.9	5.9 ± 1.6	6.9 ± 0.8	23.3 ± 5.6	9.23
Vanillic acid	71.1 ± 7.8	81.3 ± 1.5	87.8 ± 5.0	71.2 ± 3.9	89.7 ± 3.1	81.7 ± 4.1	9.5 ± 1.7	13.22
Gallic acid	14.2 ± 3.7	35.4 ± 7.0	4.6 ± 2.2	8.0 ± 1.3	9.4 ± 2.9	5.8 ± 1.1	36.2 ± 4.4	11.52
Apigenin	9.2 ± 4.0	1.3 ± 0.2	2.2 ± 0.0	1.7 ± 0.3	1.6 ± 0.3	1.6 ± 0.2	1.7 ± 0.36	4.65
Chicoric acid	36.3 ± 3.5	212.6 ± 12.3	43.3 ± 0.7	51.4 ± 2.3	108.0 ± 13.0	44.4 ± 7.6	58.7 ± 6.70	19.56
Luteolin	211.8 ± 33.8	311.3 ± 34.1	299.5 ± 46.9	46.5 ± 6.1	345.4 ± 56.1	293.2 ± 57.1	112.4 ± 23.8	146.12
Kaempferol	20.6 ± 6.2	0.5 ± 0.0	2.8 ± 0.3	8.6 ± 3.5	23.4 ± 10.1	89.8 ± 23.1	2.4 ± 1.2	87.65
2,4-Dihydroxybenzoic acid	4.9 ± 0.4	2.7 ± 0.4	4.2 ± 0.7	2.6 ± 0.2	3.2 ± 0.7	3.4 ± 0.7	7.4 ± 2.3	3.08
Naringenin	14.0 ± 1.2	10.7 ± 0.4	12.8 ± 0.5	10.3 ± 0.7	11.1 ± 0.4	10.4 ± 1.1	18.8 ± 3.8	4.80
Coumarin	65.7 ± 3.4	164.0 ± 1.2	121.3 ± 10.3	101.4 ± 2.1	193.9 ± 12.4	83.0 ± 8.9	122.9 ± 6.4	22.90

^a^Within each row means with the least significant difference (LSD test at 0.05 level) are not significantly different from each other.

### Antioxidant properties

Figure 1 shows the free radical-scavenging activities of the seven oregano accessions which were assessed using the DPPH method. According to the obtained results, there was not a high difference among the oregano accessions in terms of DPPH antioxidant activity. However, the maximum antioxidant capacity belonged to Baneh population (IC_50_ = 91.3 µg mL^−1^). It is notable to mention that the IC_50_ content (50% reduction in DPPH concentration) for ascorbic acid as the positive control was 9.95 µg mL^−1^. On the other hand, no significant difference was found among the oregano accessions in terms of DPPH antioxidant capacity. According to the mean compression, the FRAP activity of oregano accessions ranged from 347.7 to 493.6 (µmol g^−1^ DW). Overall, Gilan accession (subsp. *virens*), with 493.6 µmol g^−1^ DW, showed the highest FRAP antioxidant activity; however, there were no statistically significant differences between other investigated accessions (Fig. 1). The FRAP activity of ascorbic acid as the positive control was 1946.6 µmol g^−1^.

### Multivariate analysis of phenolic antioxidants

**Figure 2:**
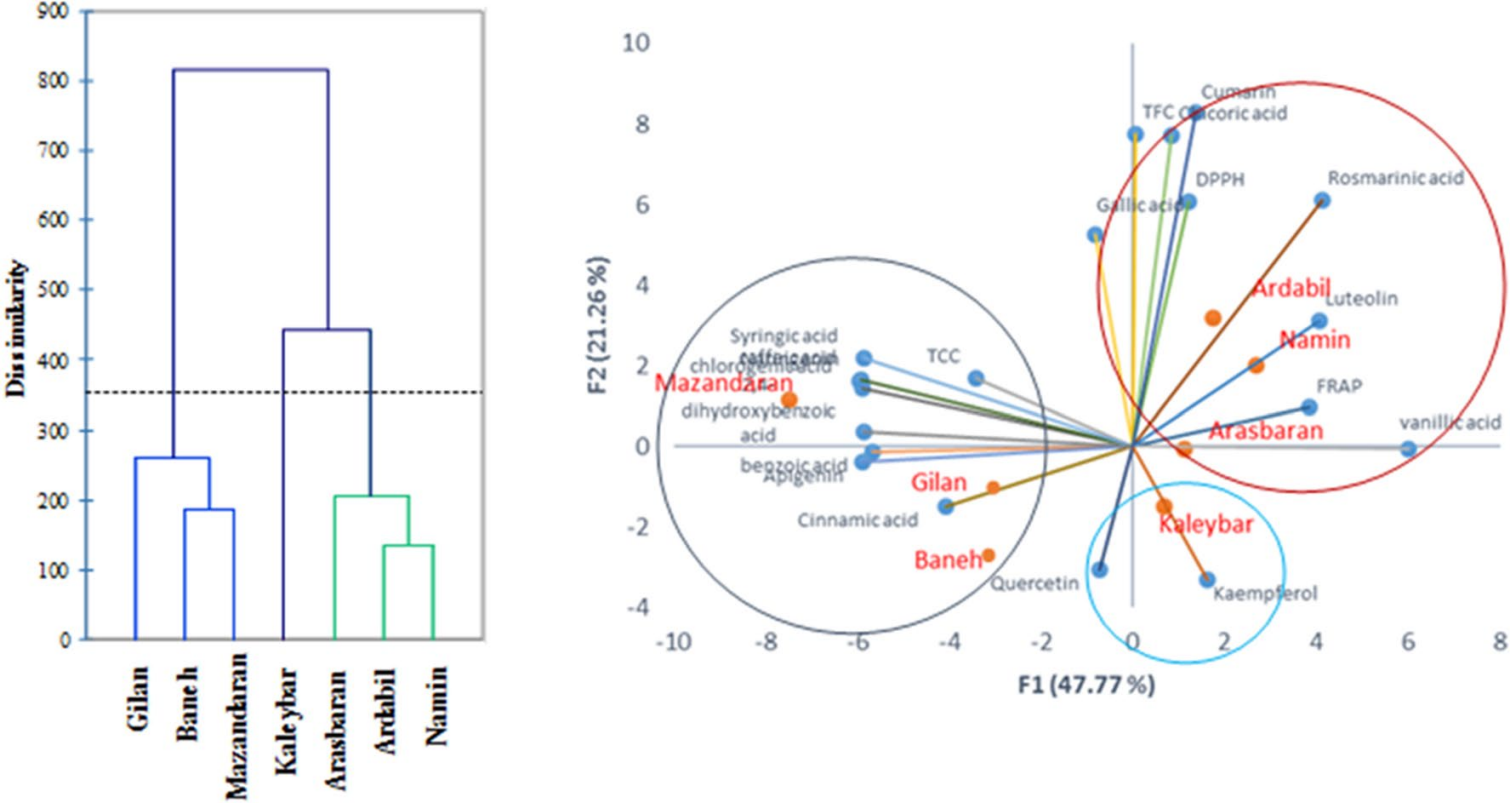
Agglomerative hierarchical clustering and principal component analysis (PCA) classification of oregano accessions based on their phenolic compounds and antioxidant properties. TPC: Total phenol content, TFC: Total flavonoids content.

Cluster analysis (CA) and principal components analysis (PCA) were performed using a data matrix composed of 133 data points (19 variables × 7 observations) to display the grouping among oregano accessions based on their phenolic components and antioxidant attributes (Fig. 2). The first two components explained 69.03% of the total variance. According to the CA and PCA analyses, the mentioned accessions were classified into three different groups (Fig. 2). The first cluster was composed of three accessions (namely, Mazandaran (subsp. *virens*), Gilan (subsp. *virens*) and Baneh (subsp. *gracile*) including the lowest rosmarinic acid and luteolin content, and DPPH radical scavenging activity, and higher amounts of apigenin. Furthermore, Kaleybar accession is in close angle with kaempferol and placed in a distinct group (Fig. 2). However, Arasbaran (subsp. *vulgare*), Ardabil (subsp. *virens*) and Namin (subsp. *virens*) accessions possessed the highest rosmarinic acid, luteolin, vanillic acid, chicoric acid, coumarin and DPPH radical scavenging activity and were placed in the same group. Simple correlation analysis showed a significant relationship between the TFC and TPC content (r = 0.58, *p* < 0.01), as well as to chicoric acid (r = 0.73, *p* < 0.01). Furthermore, correlation analysis revealed that rosmarinic acid content is moderately correlated with DPPH antioxidant activity (r = 0.46, *p* < 0.05). Moreover, there were negative correlations between vanillic acid and caffeic acid content (r = −0.92, *p* < 0.01), and syringic acid (r = −0.93, *p* < 0.01). Statistical analysis also revealed moderate and significant relationships between the chlorogenic acid and caffeic acid (r = 0.67), syringic acid and benzoic acid (r = 0.70), syringic acid and naringenin (r = 0.67) (Table 3).

**Table 3:** Pearson’s correlation coefficients among antioxidants and phenolic components of oregano accessions.

	TFC	TPC	DPPH	Rosmarinic acid	Chlorogenic acid	Cinnamic acid	Quercetin	Caffeic acid	Syringic acid	Benzoic acid	vanillic acid	Gallic acid	Apigenin	Chicoric acid	Luteolin	Kaempferol	Naringenin
TFC	1.000																
TPC	0.573**	1.000															
DPPH	0.076	0.279	1.000														
Rosmarinic acid	0.424	0.227	0.458*	1.000													
Chlorogenic acid	0.104	0.294	−0.171	−0.215	1.000												
Cinnamic acid	−0.396	−0.011	0.004	−0.157	0.033	1.000											
Quercetin	−0.001	−0.366	−0.602**	−0.403	0.034	−0.443*	1.000										
Caffeic acid	0.098	0.448*	0.006	−0.229	0.675**	−0.044	0.012	1.000									
Syringic acid	0.148	0.474*	−0.045	−0.168	0.638**	−0.036	−0.043	0.972**	1.000								
Benzoic acid	−0.121	0.278	0.047	−0.291	0.519*	−0.103	0.099	0.802**	0.703**	1.000							
Vanillic acid	−0.084	−0.361	0.188	0.407	−0.658**	0.047	−0.107	−0.925**	−0.936**	−0.609**	1.000						
Gallic acid	0.470*	0.224	0.031	0.070	0.376	−0.385	0.379	0.611**	0.599**	0.493*	−.529*	1.000					
Apigenin	−0.256	−0.372	−0.354	−0.415	−0.014	−0.338	0.499*	−0.103	−0.110	−0.131	−0.089	−0.170	1.000				
Chicoric acid	0.735**	0.073	0.208	0.616**	−0.091	−0.466*	0.101	−0.157	−0.122	−0.202	0.217	0.554**	−0.290	1.000			
Luteolin	0.206	−0.064	0.155	0.635**	−0.357	0.083	−0.108	−0.383	−0.366	−0.379	0.517*	−0.069	−0.159	0.345	1.000		
Kaempferol	−0.058	−0.115	−0.057	−0.019	−0.238	0.331	−0.116	−0.130	−0.134	−0.176	0.195	−0.232	−0.051	−0.208	0.171	1.000	
Naringenin	−0.120	0.197	−0.031	−0.263	0.466*	−0.075	0.189	0.787**	0.672**	0.922**	−0.620**	0.508*	−0.004	−0.187	−0.281	−0.163	1.000

* and **, significant difference at 5 and 1%, respectively.

## Discussion

*O. vulgare* is a popular medicinal herb, which is frequently used as antioxidant, antifungal, antimicrobial, expectorant, stimulant, carminative, anti-cancer and ant-aging agent among other applications in the food and beverage industries^7,9^. Natural antioxidants isolated from oregano extracts have been found to have many health effects such as mental well-being and decreasing postsurgical wounds infection^6,9^. The quality of oregano plants based on antioxidant efficacy is significantly influenced by subspecies and geographical origin. Such variation could be due to the plant genetics and environmental conditions, such as light intensity, soil conditions and water availability^20^. The results of the present work showed a high variation in drug yield of Iranian oregano accessions. Concerning the planting in the same controlled environmental and growing conditions, such differences in drug yield may be related to the genetic factors of oregano subspecies^15^. Genetic breeding in oregano subspecies can potentially aid in achieving superior agronomic properties, higher yields and desirable cultivars rich in phenolic compositions^5,11^. Therefore, accessions with higher biomass yield can be considered for higher production of secondary metabolites in breeding programs.

*O. vulgare* is a rich source of phenolic acids and flavonoids with a strong antioxidant activity, which qualify the plant as a potential functional food and medicine^10,11,21^. Polyphenols can fluctuate broadly in their arrangement and overall classification; however, all of them share the common structure of comprising a minimum of one aromatic ring and one or more hydroxyl groups^22^. As a matter of fact, phenolic compounds are considered as important ingredients of plants, fruits and vegetables^23^. Here, a high variation in TPC and TFC was observed among the investigated accessions belonging to three different subspecies. The highest TPC was found in Mazandaran (subsp. *virens*) accession, which was about 42% higher than that of Baneh (subsp. *gracile*) accession. Regarding flavonoids, the highest concentration was obtained from Ardabil (subsp. *virens*), while the lowest was seen in Arasbaran (subsp. *vulgare*) accession. Among these accessions, the highest TFC was about three times higher than the lowest one. In agreement with the present study, a comparative study on European oregano demonstrated that there is a high variation among the oregano accessions in terms of TPC and antioxidant activity^11^. Compared with our study, Dambolena et al. (2010)^24^ found a smaller variation in the TPC of oregano subspecies; however, they reported a range of 17 to 19.4 mg GAE/g DW for TPC that was in agreement with our findings. Yan et al. (2016)^11^ reported a higher amount of TPC (ranging from 93 to 135 mg GAE/g DW) in European oregano subspecies; however, the TPC in our subspecies is comparable to those reported by Pizzale et al. (2002)^25^ and Capecka et al. (2005)^26^. These disparate results may imply that several factors, such as extraction methods, genetic controls, environmental parameters, growing and post-harvesting conditions as well as gene expression patterns may affect plant metabolism^27^.

Oregano contains different classes of polyphenolic compounds with multiple biological effects, such as antioxidant activity^28^. In the present study, a high antioxidant activity was observed among the oregano accessions and was confirmed by the abundance of their polyphenol compounds. The highest DPPH scavenging activities was detected in the Namin accession; however, the highest FRAP activity was obtained in Gilan accession. Interestingly, both accessions belonged to the *virens* subspecies. In accordance with our results, a high variation in antioxidant activity has previously been reported for oregano accessions^11^. Consistent with our results, the study of Lamien-Meda et al. (2010)^29^ showed a similar range for DPPH antioxidant activity among the 19 sage (*Salvia officinalis* L.) accessions (ranging from 64 to 132 mg TE/g DW). However, the upper range in FRAP activity was observed in our study. The total and specific polyphenol compounds are the key accountable agents for the antioxidant activity of oregano species^30^. Previous studies on oregano showed a high antioxidant activity of dry leaves in olive oil as well as a high increase in oxidative stability of fried chips^9^.

Rosmarinic acid is one of the major phenolic acids which have been reported in the oregano plant^31^. This component is also found in other species of the Lamiaceae family, such as thyme (*Thymus daenensis* and *Thymus vulgaris*)^32^, basil (*Ocimum* spp.)^23^, mint (*Mentha* spp.)^33^ and sage (*Salvia officinalis*) (Lamien-Meda et al., 2010). In the current study, a high variation in rosmarinic acid content was found among the oregano subspecies (ranged from 659.6 to 1646.9 mg/100 g DW). An approximately similar variation (ranging from 7.2 to 41.3 mg/g DW) was previously reported in oregano accessions by Yan et al. (2016)^11^. Interestingly, based on a previous study^34^, the average content of rosmarinic acid for subspecies *vulgare* was 18.2 mg/g DW which was in good agreement with our results. This variation in rosmarinic acid content provides valuable data for oregano breeders for achieving high quality plants with high antioxidant capacity for food and medicinal applications. Results also demonstrate that the main bioactive component of accession with the lowermost to the uppermost concentration varied among the oregano accessions, for instance about 6.6-fold for luteolin, tenfold for quercetin, threefold for coumarin and 5.8-fold for chicoric acid (Table 2). Our findings agree with those of others, specifying that oregano species were reported to be rich sources of phenolic acids such as rosmarinic acid^11^. In accordance with our results on phenolics, literature review illustrates the existence of diverse phenolic constituents in the *Oregano* species, such as rosmarinic acid, hydroxycinnamic acid, apigenin, luteolin, quercetin, scutellarein, apigenin-7-O-glucoside, luteolin-7-O-glucoside, and luteolin-7-O-glucuronide^11,18,35^. The composition of phenolic compounds in oregano depends on the accession, subspecies, genotype, and geographical and environmental conditions. It has been shown that the flavonoid and phenolic compounds of oregano can be used to differentiate between oregano chemotypes within the same species. For instance, the concentration of rosmarinic acid has been reported to vary between the chemotype within the species of *O. vulgare* subp. *hirtum*, *O. vulgare* subsp. *vulgare* and *O. syriacum*^34^.

Correlation analysis showed some relations among phenolic compounds and antioxidant attributes. However, there was not a significant correlation among the TFC and antioxidant activity. The results demonstrated a moderate correlation among DPPH antioxidant activity and rosmarinic acid, but no significant correlation was observed among FRAP activity and rosmarinic acid content. Similar to our results, Yan et al. (2016)^11^ reported that there is no correlation between the antioxidant capacity measured by oxygen radical absorbance capacity and the concentration of rosmarinic acid. It can be specified that other phenolics and constituents can also be considered as antioxidant agents. In another study, the authors ascribed the antioxidant capacity of the similar fairly extract to the amount of rosmarinic acid and other bioactive polyphenol constituents^36^.

## Conclusion

Oregano is a widely used medicinal herb due to its special phenolic compounds, aroma transfer and medicinal properties. In this study, the genetic variation in phenolic components and antioxidant activity of Iranian oregano accessions was investigated. A high variation was observed between the highest and lowest quantity of concentration of rosmarinic acid and other phenolics. These results might be utilized for breeding oregano plants containing high amount of phenolic components and exhibiting high antioxidant activity. Upgraded species and accessions with great polyphenolic compounds and small progressive duration is of high interest to plant producers. The extracts of herbs as well as other plant materials are gradually gaining attention in numerous industrial applications based on natural products. As a result, identification of different accessions which are richest in special components is very important for food and pharmaceutical industries. Finally, the survey of our study offers a broad-ranging variability among the Iranian oregano populations for utilizing elite accessions and subspecies (especially Ardabil and Namin accessions belonging to *virens* subspecies) based on phenolic components and antioxidant attributes for breeding programs and domestication purposes.
